# The Relationship Between the Use of Speech-Generating Devices and Verbal Requests in Children with Autism Spectrum Disorder and Intellectual Disabilities

**DOI:** 10.1007/s10803-025-06910-z

**Published:** 2025-06-07

**Authors:** Moeka Kaneda, Ginga Sasaki

**Affiliations:** 1 Graduate School of Comprehensive Human Sciences, University of Tsukuba, Tsukuba, Ibaraki Japan; 2 Present Address: Niigata-shiritsu Higashi Schools for Special Needs Education, Niigata, Niigata, Japan; 3 Institute of Human Sciences, University of Tsukuba, Tsukuba, Ibaraki Japan

**Keywords:** Speech-generating Devices (SGDs), Augmentative and Alternative Communication (AAC), Autism Spectrum Disorder (ASD), Intellectual Disabilities, Verbal Requests

## Abstract

**Purpose:**

This study aimed to investigate the effects of removing synthetic speech output from speech-generating devices (SGDs) and incorporating delayed reinforcement on verbal requests in children with autism spectrum disorder (ASD) and intellectual disabilities. Additionally, we examined how participants’ receptive and expressive language abilities influenced vocal requests by comparing items categorized as easier or more difficult to name based on pre-assessment results.

**Methods:**

Three children with ASD and intellectual disabilities participated. An alternating treatment design was used to compare two conditions: (1) synthetic speech output with immediate reinforcement and (2) no synthetic speech output with delayed reinforcement. Preferred items were classified into two sets based on pre-assessed receptive and expressive language abilities. The dependent variable was the number of verbal and SGD-based requests.

**Results:**

Results showed that verbal requests increased in the no synthetic speech output with delayed reinforcement condition across both item sets, while SGD-based requests remained high. Post-intervention language assessments indicated significant improvements in expressive language abilities.

**Conclusion:**

These findings suggest that removing synthetic speech output and incorporating delayed reinforcement may effectively promote vocal requests in children with ASD and intellectual disabilities. This intervention provides a novel approach to facilitate speech communication while continuing the use of AAC tools.

## Introduction

It is estimated that approximately 30% of individuals with Autism Spectrum Disorder (ASD) never develop functional speech (Lorah et al., 2022). For children with ASD who exhibit these characteristics, Augmentative and Alternative Communication (AAC) strategies have been recommended to facilitate functional communication (Mirenda, 2003).

One type of AAC is the Speech-Generating Device (SGD), an electronic communication tool. SGDs produce speech output when users select icons, pictures, or words displayed on the screen (Lorah et al., 2015). As electronic tablets, such as the Apple iPad®, are increasingly utilized as SGDs (Lorah et al., 2022), their adoption as AAC tools has expanded. SGDs offer the advantage of generating consistent speech output and facilitating message interpretation for listeners (Alzrayer et al., 2020). Also, managing all communication symbols is straightforward because they are stored within the device. However, tapping an SGD does not inherently function as a linguistic behavior (Bondy, 2012). If tapping the device occurs at a similar frequency regardless of whether a listener is present, the SGD may serve more as a toy than as a communication tool (Bondy, 2019). Therefore, it is crucial to determine whether the synthetic speech generated by SGDs functions as communication rather than mere sound output (Bondy, 2019).

Interventions involving SGDs have shown promise in promoting vocalizations and speech (Yuan et al., 2024). Yuan et al. (2024) conducted a systematic review of studies utilizing SGDs to enhance vocalizations and speech in children with ASD and complex communication needs. The review included 14 single-case experimental design studies and 4 group design studies published between 1990 and 2023. Among 108 participants, 95 (87%) exhibited improvements in vocalizations and speech to varying degrees. Kasari, Kaiser, Goods, Nietfeld, Mathy, Landa, Murphy, and Almirall (2014) suggested that SGD use may facilitate speech acquisition by providing consistent vocal models, increasing their frequency, and presenting symbols alongside speech output on the device screen.

These findings suggest that the synthetic speech output of SGDs may contribute to facilitating vocal requests. Previous studies have investigated the impact of the presence or absence of synthetic speech output from SGDs on vocal requests (Schlosser, Sigafoos, Luiselli, Angermeier, Harasymowyz, Schooley, & Belfiore, 2007; Sigafoos et al., 2003). However, none of these studies identified a significant effect of synthetic speech output. Notably, in conditions where synthetic speech was absent, SGD-based requests were immediately reinforced. This immediate reinforcement may have strengthened SGD-based requesting behaviors, thereby reducing the likelihood of vocal requests.

An additional intervention method involves delaying the presentation of reinforcers to promote vocal requests while utilizing SGDs. Studies have indicated that incorporating delayed reinforcement into interventions facilitates both SGD-based requesting and vocal requests (Alzrayer et al., 2020; Bishop, Moore, Dart, Radley, Brewer, Barker, Quintero, Litten, Glifeather, Newborne, & Toche, 2020; Gevarter & Horan, 2019; Gevarter, O’Reilly, Kuhn, Mills, Ferguson, Watkins, Sigafoos, Lang, Rojeski, & Lancioni, 2016). This approach is often combined with other strategies, such as vocal prompting and differential reinforcement (Gevarter & Horan, 2019). For instance, Gevarter et al. (2016) conducted an intervention targeting vocal requests in 4 children with ASD, integrating SGD-based requesting with delayed reinforcement and differential reinforcement of vocal requests. As a result, 2 participants demonstrated increased approximations of target words.

Although studies have examined the effects of synthetic speech output and additional interventions on vocal requests, few have evaluated participants’ ability to comprehend and express the names of items used in interventions. While many studies on SGD and vocal requests have assessed participants’ vocal imitation or expressive language skills (Yuan et al., 2024), few have pre-assessed the extent to which participants understand and articulate the names of items. Although some studies have conducted naming evaluations before and after interventions (Brady, Storkel, Bushnell, Barker, Saunders, Daniels, & Fleming, 2015; Schlosser et al., 2007), the frequency of vocalizations in real-life requesting situations remains largely unexamined.

Thus, previous studies have not explored how receptive and expressive language variations influence the frequency of vocalizations in real-life requesting scenarios. Assessing participants’ receptive and expressive language abilities related to item names before an intervention could provide insight into whether existing stimulus relationships are reinforced during vocal requests or if new associations emerge through the use of SGD.

This study had two primary objectives. First, we aimed to investigate the effects of removing synthetic speech output from SGDs and incorporating delayed reinforcement on verbal requests in children with ASD and intellectual disabilities. Second, we compared and analyzed the results based on participants’ pre-assessed receptive and expressive language abilities related to item names, specifically distinguishing between easier or more challenging items to name.

## Method

### Participants

The participants were three children with ASD and intellectual disabilities: Ken, Hana, and Ryo. Their medical diagnoses were confirmed by diagnostic reports and/or official opinion. They did not exhibit any challenging behaviors.

Ken was an 11 years 7 months old boy enrolled in the elementary division of a special needs school at the start of the study. The Kyoto Scale of Psychological Development 2020 (Koyama et al., 2009), administered at 11 years and 6 months of age, indicated a cognitive-adaptive ability of 3 years and 7 months (Developmental Quotients = DQ31), language-social ability of 3 years and 6 months (DQ31), and an overall developmental level of 3 years and 7 months (DQ31). At the beginning of this study, Ken was able to point to and request desired items using one- to two-word phrases, such as “**●●**” or “○○-SURU (do ○○).” Regarding the history of AAC use, Ken had received PECS^®^ (Picture Exchange Communication System; Bondy & Frost, 2001) instruction in a university-based tutoring setting. By 11 years and 4 months of age, he could independently use PECS^®^ Phase IV without prompting. Regarding his history of SGD use, Ken had received instruction in SGD-based requesting during university tutoring sessions. By 10 years and 2 months of age, he was able to tap a photo button to request a preferred item from a set of six options. However, the SGD application used in this study differed from the one he had previously used.

Hana was a 7 years 7 months old girl enrolled in the elementary division of a special needs school at the start of the study. The Kyoto Scale of Psychological Development 2020, administered at 7 years and 6 months of age, assessed her cognitive-adaptive ability at 3 years and 3 months (DQ43), language-social ability at 3 years and 3 months (DQ43), and overall developmental level at 3 years and 4 months (DQ44). At the beginning of this study, Hana was able to pull others’ hands to gain attention and request desired items using one- to two-word phrases, such as “**●●**” or “○○-CHODAI (please give me ○○).” Regarding her history of AAC use, Hana received PECS^®^ instruction at 7 years and 4 months of age in a university-based tutoring setting and successfully acquired PECS^®^ Phase III. Hana had prior experience using a tablet but had not used an SGD as a communication tool.

Ryo was a 12 years 1 month old boy enrolled in the elementary division of a special needs school at the start of the study. The Kyoto Scale of Psychological Development 2020, administered at 12 years of age, assessed his cognitive-adaptive ability at 3 years and 5 months (DQ28), language-social ability at 3 years and 0 months (DQ25), and overall developmental level at 3 years and 3 months (DQ27). At the beginning of the study, Ryo was able to point and request desired items using one- to two-word phrases, such as “**●●**” or “OCHA-NOMU (drink tea).” Regarding his history of AAC use, Ryo received PECS^®^ instruction at 11 years and 10 months of age in a university-based tutoring setting and successfully acquired PECS^®^ Phase III. Ryo had prior experience using a tablet but had not used an SGD as a communication tool.

### Setting and Materials

In principle, the SGD intervention was conducted one to four times per day, with each session lasting 10 to 20 min during a one-hour tutoring session held once per week at University of Tsukuba. The SGD was placed on a desk in front of the participants while the items, which were preferred objects for them as listed in Table 1, were arranged on a shelf approximately 150 cm in front of them, making the items clearly visible yet physically out of reach. The distance between participants and SGD was approximately 15 to 20 cm. The experimenter (1st author) or an assistant provided the items.

**Table 1:** The names of the items used by each participant

Ken		Hana		Ryo	
Naming Possible Set	Naming Difficult Set	Naming Possible Set	Naming Difficult Set	Naming Possible Set	Naming Difficult Set
INU (Dog Doll)	ANPANMAN-NO-TAIKO (Anpanman Drum)	ANPANMAN (Anpanman Capsule Toy)	DOUBUTSU-NO-JIGSAW-PUZZLE (Animal Jigsaw Puzzle)	KOMA (Spinning Tops)	ANPANMAN-NO-EHONAN (Anpanman Picture Book)
OUTA-EHON (Singing Picture Book)	OHAJIKI (Ohajiki)	KOROKORO (Kugelbahn)	NORIMONO-NO-JIGSAW-PUZZLE (Vehicle Jigsaw Puzzle)	THOMAS (Thomas Picture Book)	ANPANMAN-NO-PUZZLE (Anpanman Puzzle)
PIANO-EHON (Piano Picture Book)	DOUBUTU-NO-TAIKO (Animal Drum)	TOKEI (Clock Puzzle)	MIDORI-NO-DOUBUTSU-NO-PUZZLE (Green Animal Puzzle)	TOKEI (Clock Puzzle)	OSHIRITANTEI-NO-EHON (Oshiritantei Picture Book)
HIYOKO (Chick Doll)	HAND-SPINNER (Hand Spinner)	HELLO-KITTY (Hello Kitty Flash Spin Light)	KIIRO-NO-NORIMONO-NO-PUZZLE (Yellow Vehicle Puzzle)	HIYOKO (Chick Doll)	OSHIRITANTEI-NO-PUZZLE (Oshiritantei Puzzle)
PUCHIPUCHI (Push Pop)	MIDORI-NO-TAIKO (Green Drum)	HOIPP (Whipped Cream Cake Toy)	HIRAGANA-NO-PUZZLE (Hiragana Puzzle)	BUTA (Pig Sound Picture Book)	KOROKORO (Kugelbahn)
YO-YO (Yo-Yo)	ROBOTTO (Robot Toy)	RAMEN (Ramen Toy)	HIRAGANA-CARD (Hiragana Cards)	PENGIN (Penguin Toy)	BLOCK (Shape Sorter Toy)

The SGD was implemented using an Apple iPad mini^®^ running the “DropTalk” application. The iPad mini was equipped with a protective cover, which, when opened, revealed a photo button screen displaying images of items.

### Experimental Design

This study employed an alternating treatment design to compare the naming possible set and naming difficult set conditions with an ABA design. In this design, condition A involved speech output being on with immediate reinforcement, while condition B involved speech output being off with delayed reinforcement.

### Dependent Variable

The dependent variable was the number of requests made through verbalization or SGD (times).

### Procedure

#### Preference Assessment

Using the Reinforcement Assessment for Individuals with Severe Disabilities (RAISD; Fisher et al., 1996), parents responded to a questionnaire. In addition to the questionnaire responses, at least 12 preferred items were identified based on parent interviews and observations of the participants during their free time (approximately 10–15 min) in the tutoring sessions at University of Tsukuba.

#### Pre-Language Assessment

The participants’ receptive and expressive language abilities were assessed for items identified as preferred in the preference assessment.

First, regarding receptive language, the experimenter presented six picture cards representing the participants’ preferred items and asked, “Which one is ●●?” If the participant selected the correct card corresponding to the item named by the experimenter, it was considered a correct response and was followed by verbal praise. However, the item itself was not provided. If the participant gave an incorrect response or failed to respond (i.e., no response for more than five seconds), the experimenter proceeded to the next trial.

Next, for the expressive language task, the experimenter presented one photo card at a time and asked, “What is this?” If a third party could correctly identify the item based on the participant’s response, it was considered a correct response. However, if the third party could not identify the item, even if the utterance seemed to refer to the item, the response was considered incorrect. For example, if the participant said “TAIKO (drum) " for the item “MIDORI-NO-TAIKO (green drum),” the response was considered incorrect because it could also refer to other items, such as “ANPANMAN-NO-TAIKO (Anpanman drum)” or “DOUBUTSU-NO-TAIKO (animal drum).” In the case of a correct response, verbal praise was given, but the item itself was not provided. If the response was incorrect or no response occurred (i.e., no response for more than five seconds), the experimenter proceeded to the next trial.

Four trials were conducted per item for both receptive and expressive language tasks. The “naming possible” set was defined as items for which participants gave three or more correct responses in both receptive and expressive language trials. The “naming difficult” set was defined as items for which participants gave three or more correct responses in the receptive language trials but one or fewer correct responses in the expressive language trials. The language assessment continued until six items were selected for the naming possible set (indicating that both language reception and expression were possible), and six items were selected for the naming difficult set (indicating that language expression was difficult, but language reception was possible). Table 1 shows the names of the items used by the experimenter for each participant, as determined by the pre-language assessment.

#### Pre-SGD Training

The instruction for requesting using the SGD was conducted at a desk. After the participant opened the cover of the SGD and demonstrated the behavior of tapping the photo button, the corresponding item was presented to them for a match check. A correct response was defined as when the selected photo button matched the actual item, and the corresponding item was then offered to Ken for approximately 30 s and to Hana and Ryo for approximately 1 min. The six items selected in the pre-language assessment were used for each condition. Five trials were conducted in one session, and the achievement criterion was a correct response rate of 80% or higher for three consecutive sessions. After instruction with the naming possible set, the same procedure was applied to the naming difficult set.

#### Speech Output on and Immediate Reinforcement

In each session, one item from the naming possible set or naming difficult set was used. Initially, sessions began with the naming possible set, followed by randomized implementation of either set across subsequent sessions. The actual items in the selected set were placed within the participant’s view but out of reach. The SGD was positioned in front of the participant, displaying images of the selected set within the application. To establish a request opportunity, the experimenter verbally instructed the participant: *“*Tell me one toy you want.*”* A verbal request was considered correct if the participant’s utterance corresponded to an item in the presented set and was identifiable by a third party. Therefore, for example, if the item was “MIDORI-NO-TAIKO (green drum),” utterances such as “MIDORI-TAIKO (green drum)” or “MIDORI (green)” were also considered correct responses. For SGD-based requests, a correct response occurred when the participant opened the cover and tapped the photo button corresponding to the item on the SGD screen in front of the experimenter. In both cases, when a correct response occurred, the experimenter provided feedback by repeating the item’s name and then delivering the item. If an incorrect response was made (e.g., other requesting behaviors or utterances that did not allow a third party to identify the item) or if no response occurred within 10 s, the experimenter proceeded to the next trial. The item was delivered either by the experimenter or an experimental assistant. Participants progressed to the next condition when both verbal and SGD-based requests remained at a stable level (i.e., the difference between the maximum and minimum number of responses was less than two) for three consecutive sessions, with each session consisting of six trials. However, for Ken’s second speech output on an immediate reinforcement condition, more than three sessions were conducted due to insufficient confirmation of the transition criteria.

#### Speech Output Off and Delayed Reinforcement

The SGD was set so that tapping the photo button did not produce synthetic speech output. Additionally, the item was provided with a delay of approximately 5 s after tapping the photo button on the SGD screen. The item was promptly provided if the participant produced an utterance corresponding to the item in front of the experimenter within 5 s. If no corresponding utterance occurred within 5 s, the experimenter provided the item after giving auditory feedback by stating its name. Otherwise, the procedure remained the same as in the speech output on an immediate reinforcement condition.

#### Post-Language Assessment

A post-language assessment of the names of the items used in the experiment was conducted. The procedure was the same as the pre-language assessment.

### Interobserver Agreement

Interobserver agreement was calculated for the number of correct responses in each language assessment, the number of requests made verbally or via SGD in each condition, and the specific items requested. Agreement was assessed for 50% of all trials in each language assessment and at least 33.3% of all trials in each condition for each participant. The author and a graduate student specializing in disability science independently reviewed and recorded observations from video footage of each participant. The interobserver agreement rate was calculated separately for the naming possible set and the naming difficult set using the following formula: interobserver agreement rate (%) = number of trials agreed between observers/total number of trials of the participant x 100.

Results showed that Ken had 100% agreement in all cases. Hana had 91.7% agreement in the post-expressive language assessment for the naming possible set and 100% agreement in all other cases. Ryo was 100% in agreement in all cases.

### Ethical Considerations

This study was conducted with the approval of the Research Ethics Committee in the University of Tsukuba (No. 2024–41 A). We obtained written consent for participation in the study from the parents of the participants. Behavioral assent was confirmed prior to each session: the session proceeded only if the participant voluntarily approached the session materials or reached toward them; if no approach (or avoidance behavior) was observed, the session was postponed.

## Result

### Request Behaviors Via Verbalization or SGD

#### Naming Possible Set

**Fig. 1:**
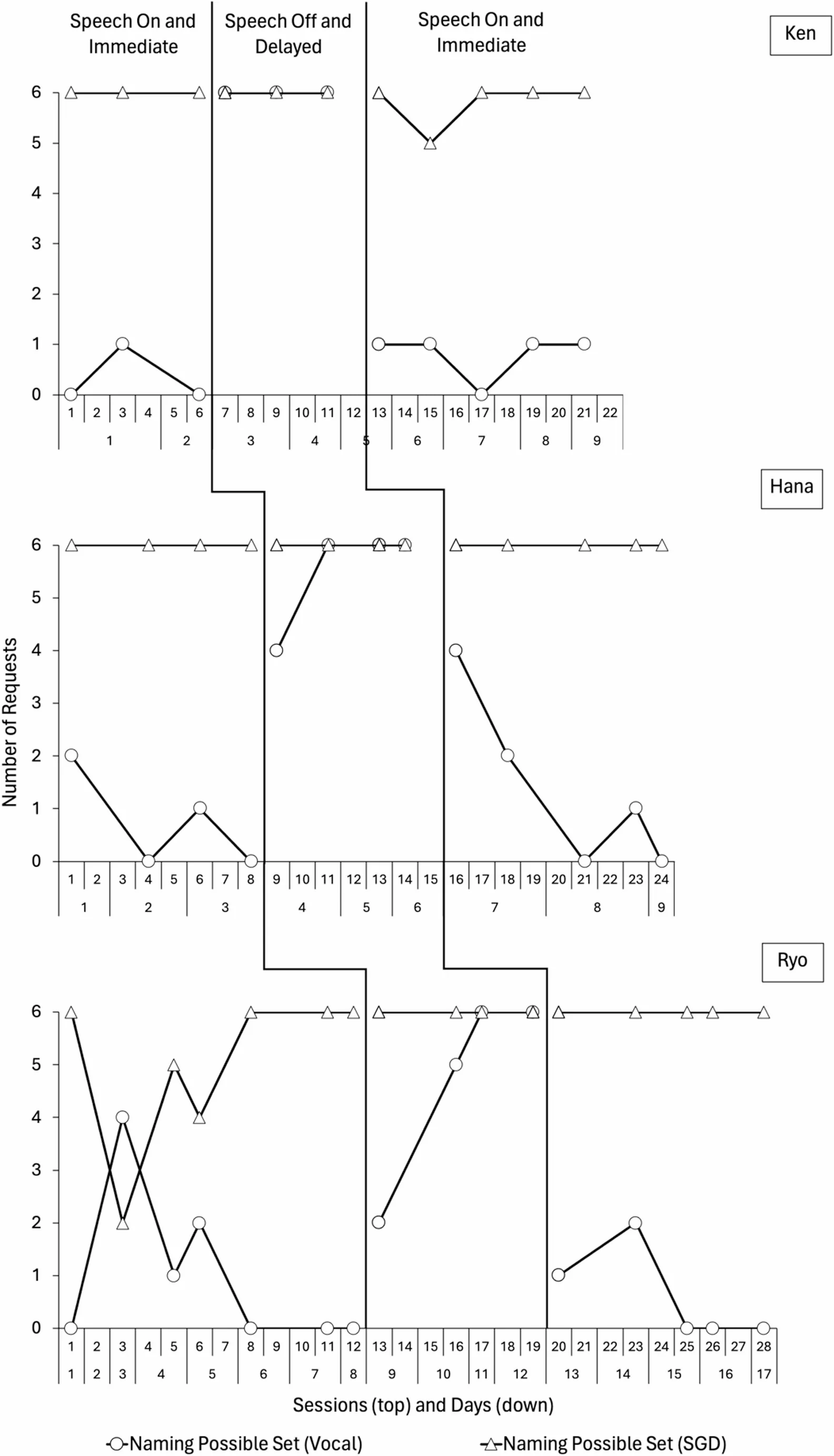
The number of requests made through verbalization or SGD in the Naming Possible Set

The number of requests made through verbalization or SGD is shown in the Naming Possible Set in Fig. 1.

For Ken, as shown in Fig. 1, verbal requests during the first speech output and immediate reinforcement condition occurred once in the third session, but no requests were observed in other sessions, with an average of 0.3 requests per session. Verbal requests were observed following the synthetic speech output of the SGD. SGD requests were more frequent than verbal requests, averaging six requests per session.

During the speech output off and delayed reinforcement condition, verbal requests increased compared to the speech output on and immediate reinforcement condition, averaging six requests per session. Verbal requests were observed either during the delay period following a photo button tap on the SGD or immediately after or simultaneously with the tap. Requests made using the SGD remained unchanged from the first speech output on an immediate reinforcement condition, averaging six requests per session. In the seventh session, during the delay period following an SGD tap, Ken exhibited repetitive tapping of the same photo button multiple times. Additionally, in the ninth session, he tapped multiple photo buttons—either clockwise or randomly—three times. After tapping the final photo button, or simultaneously with the tap, he produced a verbal request corresponding to the item.

Regarding the second speech output on an immediate reinforcement condition, the criteria for ending the condition were met during session 18. However, the experimenter had not sufficiently reviewed the criteria, leading to additional sessions being conducted. Verbal requests occurred an average of 0.8 times per session. These verbal requests were observed following the synthetic speech output from the SGD or while tapping the photo button while vocalizing. Requests made using the SGD occurred an average of 5.8 times per session. In session 15, on one occasion, after randomly tapping multiple photo buttons on the SGD, a verbal request corresponding to the last tapped item was observed. Additionally, in session 15, on one occasion, the participant repeatedly tapped multiple photo buttons for more than 10 s, and the trial was terminated.

Regarding Hana, as shown in Fig. 1, verbal requests occurred an average of 0.8 times per session during the first speech output on an immediate reinforcement condition. These verbal requests were observed simultaneously by tapping the photo button on the SGD. Requests made using the SGD were more frequent, with an average of six occurrences per session.

During the speech output off and delayed reinforcement condition, verbal requests showed an increasing trend, reaching six requests in session 11, and remained stable at that level. The average number of verbal requests was 5.5 per session. These verbal requests were observed either during the delay period after tapping the photo button on the SGD, immediately after tapping, or simultaneously with the tap. In session 9, Hana vocalized “OTOGA (sound),” grabbed the experimenter’s arm, and pulled it toward the SGD. Requests made using the SGD remained frequent, with an average of six occurrences per session, similar to the first speech output on an immediate reinforcement condition. In session 9, Hana exhibited behavior where multiple photo buttons were tapped during the delay period following the SGD tap.

Regarding the second speech output on an immediate reinforcement condition, verbal requests showed a decreasing trend. In session 16, four verbal requests were observed, but the frequency gradually declined, and by session 21, no verbal requests were made. The average number of verbal requests was 1.4 per session. These verbal requests were observed either immediately after or simultaneously with the tapping of the photo button on the SGD or while vocalizing while tapping the photo button. Requests made using the SGD remained frequent, with an average of six occurrences per session.

Regarding Ryo, as shown in Fig. 1, during the first speech output on an immediate reinforcement condition, verbal requests occurred more frequently than requests made using the SGD in session 3. However, no verbal requests were observed in sessions 8, 11, and 12. The average number of verbal requests was 1.0 per session. SGD requests were more frequent than verbal requests in all sessions except session 3, with an average of 5.0 requests per session.

During the speech output off and delayed reinforcement condition, verbal requests showed an increasing trend. In session 13, two verbal requests were observed, increasing to five in session 16, and six requests occurred in sessions 17 and 19. The average number of verbal requests was 4.8 per session. These verbal requests were observed either during the delay period after tapping the photo button on the SGD or immediately after tapping. Additionally, after tapping the photo button for the item “KOMA (spinning tops)” on the SGD, Ryo vocalized “KOROKORO (kugelbahn).” This was an instance of a vocalization that did not correspond to the item selected on the SGD. On another occasion, after tapping the photo button for “PENGIN (penguin toy),” he first vocalized “HIYOKO (chick doll)” and later corrected himself by vocalizing “PENGIN.” Requests made using the SGD were observed in all trials, with an average of six requests per session.

Regarding the second speech output on an immediate reinforcement condition, verbal requests occurred once in session 20 and twice in session 23, but no requests were made in session 25, and this trend continued with no verbal requests. The average number of verbal requests was 0.6 per session. These verbal requests were observed immediately after tapping the photo button on the SGD or after the synthetic speech output from the SGD. Requests made using the SGD were frequent, with an average of six requests per session.

#### Naming Difficult Set

**Fig. 2:**
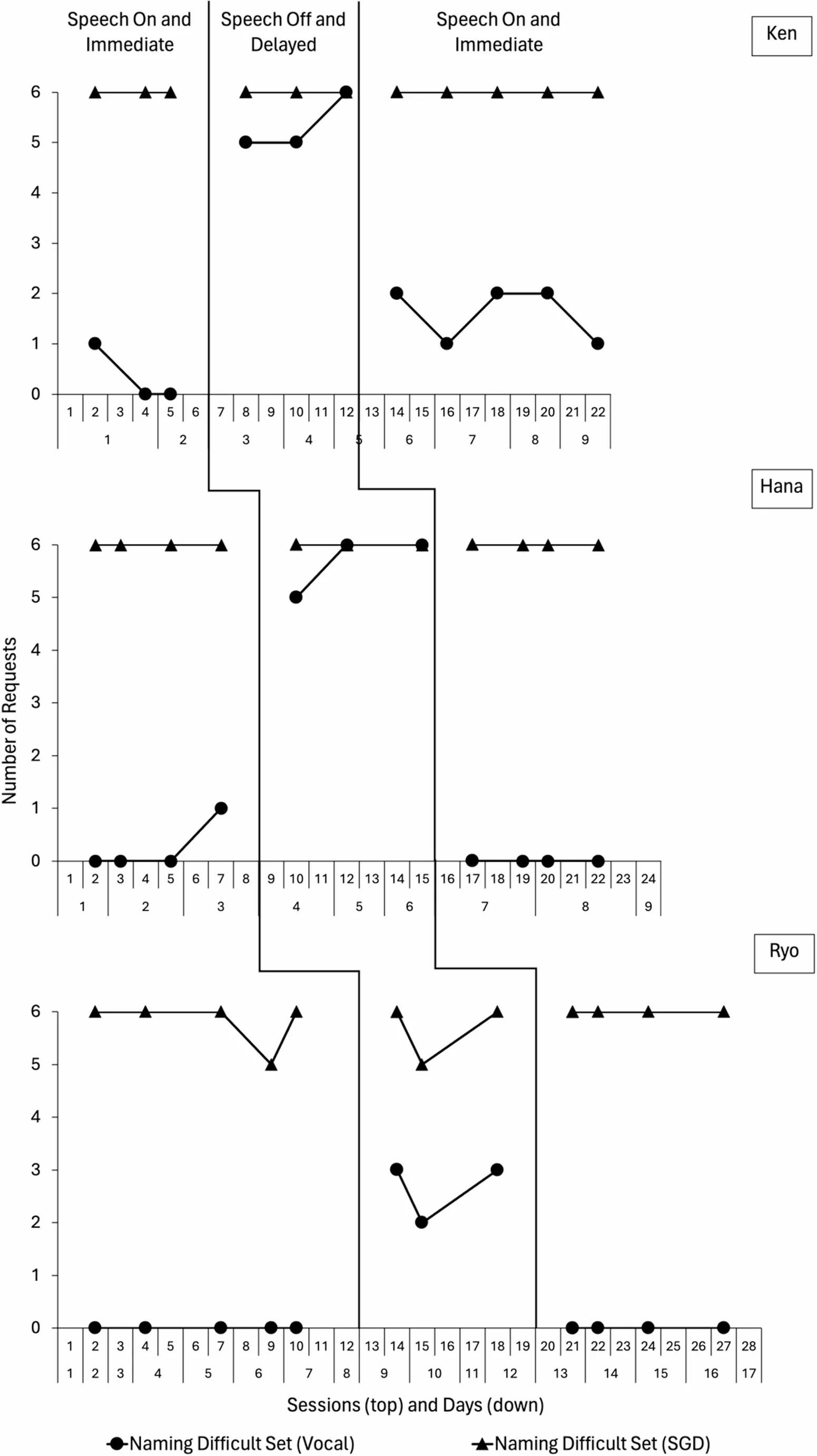
The number of requests made through verbalization or SGD in the Naming Difficult Set

The number of requests made through verbalization or SGD is shown in the Naming Difficult Set in Fig. 2.

Regarding Ken, as shown in Fig. 2, during the first speech output on an immediate reinforcement condition, verbal requests were observed once in session 2. However, no requests were made in the other sessions, resulting in an average of 0.3 verbal requests per session. These verbal requests were observed following the synthetic speech output from the SGD. Requests made using the SGD occurred more frequently, with an average of 6 requests per session.

During the speech output off and delayed reinforcement condition, verbal requests were more frequent than in the speech output on and immediate reinforcement condition, with an average of 5.3 requests per session. These verbal requests were observed either during the delay period following tapping a photo button on the SGD, immediately after tapping, or simultaneously with the tap. Additionally, after tapping the photo button for the item “ROBOTTO (robot toy)” on the SGD, verbalizations such as “KUDASAI (please give me)” or “KASHITE (please lend me)” were observed. Requests made using the SGD remained consistent with the speech output on an immediate reinforcement condition, with an average of 6 requests per session. In session 12, three instances of tapping multiple photo buttons in succession were observed. After tapping the final photo button, or simultaneously with the tap, a verbal response corresponding to the item was observed.

Regarding the second speech output on an immediate reinforcement condition, the criteria for ending the condition were met during session 18. However, the experimenter had not sufficiently reviewed the criteria, leading to additional sessions being conducted. Verbal requests fluctuated between 1 and 2 requests per session, with an average of 1.6 requests per session. These verbal requests were observed following the synthetic speech output from the SGD. Requests made using the SGD occurred an average of 6 requests per session. In session 14, once, after randomly tapping multiple photo buttons on the SGD, a verbal response corresponding to the last tapped item was observed.

Regarding Hana, as shown in Fig. 2, during the first speech output on an immediate reinforcement condition, verbal requests were observed only once in session 7, with no verbal requests in other sessions, resulting in an average of 0.3 verbal requests per session. When verbal requests occurred, they were observed before tapping the photo button on the SGD. Requests made using the SGD were more frequent, with an average of 6 requests per session.

During the speech output off and delayed reinforcement condition, verbal requests increased compared to the speech output on and immediate reinforcement condition, with an average of 5.7 requests per session. These verbal requests were observed either during the delay period after tapping the photo button on the SGD, immediately after tapping, or simultaneously with the tap. There was an instance where Hana verbalized “JIGSAW-PUZZLE,” but this was not considered a valid request as the third party could not identify the item, and thus, it was not included as a request in this study. Additionally, after verbalizing “JIGSAW-PUZZLE,” she rephrased it as “DOUBUTSU-NO-JIGSAW-PUZZLE (animal jigsaw puzzle)” twice. Moreover, after tapping the photo button for the item “MIDORI-NO-DOUBUTSU-NO-PUZZLE (green animal puzzle)” on the SGD, Hana verbalized “DOUBUTSU-NO-JIGSAW-PUZZLE.” However, she then corrected herself by verbalizing “MIDORI-NO-DOUBUTSU-NO-PUZZLE” once. Requests made using the SGD were frequent, with an average of 6 requests per session, similar to the first speech output on an immediate reinforcement condition.

Regarding the second speech output and immediate reinforcement, verbal requests decreased, with an average of 0 requests per session. In session 17, Hana verbalized “NORIMONO (vehicles)” while tapping the photo button for the item “NORIMONO-NO-JIGSAW-PUZZLE (vehicle jigsaw puzzle)” on the SGD but stopped verbalizing when the synthetic speech was output. Requests made using the SGD occurred an average of 6 requests per session.

Regarding Ryo, as shown in Fig. 2, no verbal requests were made during the first speech output on an immediate reinforcement condition, with an average of 0 requests per session. Although Ryo verbalized terms like “OSIRITANTEI (Names of Japanese characters),” “OSHIRI,” and “PUZZLE,” these were not considered valid requests as the third party could not identify the items, and thus they were not included as requests in this study. Requests made using the SGD occurred 6 times in all sessions except session 9, with an average of 5.8 requests per session. In session 9, during the 6th trial, Ryo opened the SGD cover, looked at the screen, and then closed the cover while verbalizing “OSHIMAI (finished).” As no item was requested and more than 10 s passed, there were only 5 trials in which requests occurred during session 9.

During the speech output off and delayed reinforcement condition, verbal requests fluctuated between 2 and 3 occurrences per session, with an average of 2.7 requests. These verbalizations were observed either during the delay period after tapping the photo button on the SGD or immediately after tapping. There were instances where Ryo verbalized words such as “EHON” (“picture Book”), “PUZZLE,” “OHIRITANTEI,” “OSHIRI,” and “ABC.” However, as these words could not be identified as corresponding to specific items, they were not included as valid requests in this study. Additionally, after verbalizing “ANPANMAN (Names of a Japanese character),” Ryo rephrased it as “ANPANMAN-NO-HON” (“Anpanman book”) once and as “ANPANMAN-NO-PUZZLE” (“Anpanman puzzle”) once. Requests made using the SGD occurred 6 times in all sessions except session 15, with an average of 5.7 requests per session. In session 15, during the 6th trial, Ryo verbalized “DAME (no)” and “OSHIMAI.” As no item was requested and more than 10 s passed, there were only 5 trials in which requests occurred during session 15.

Regarding the second speech output on an immediate reinforcement condition, verbal requests decreased, with an average of 0 requests per session. Although Ryo verbalized “OSHIRITANTEI” once, this was not considered a valid request as it could not be identified as corresponding to a specific item, and thus, it was not included as a request in this study. Requests made using the SGD occurred an average of 6 requests per session.

### Language Assessment

**Fig. 3:**
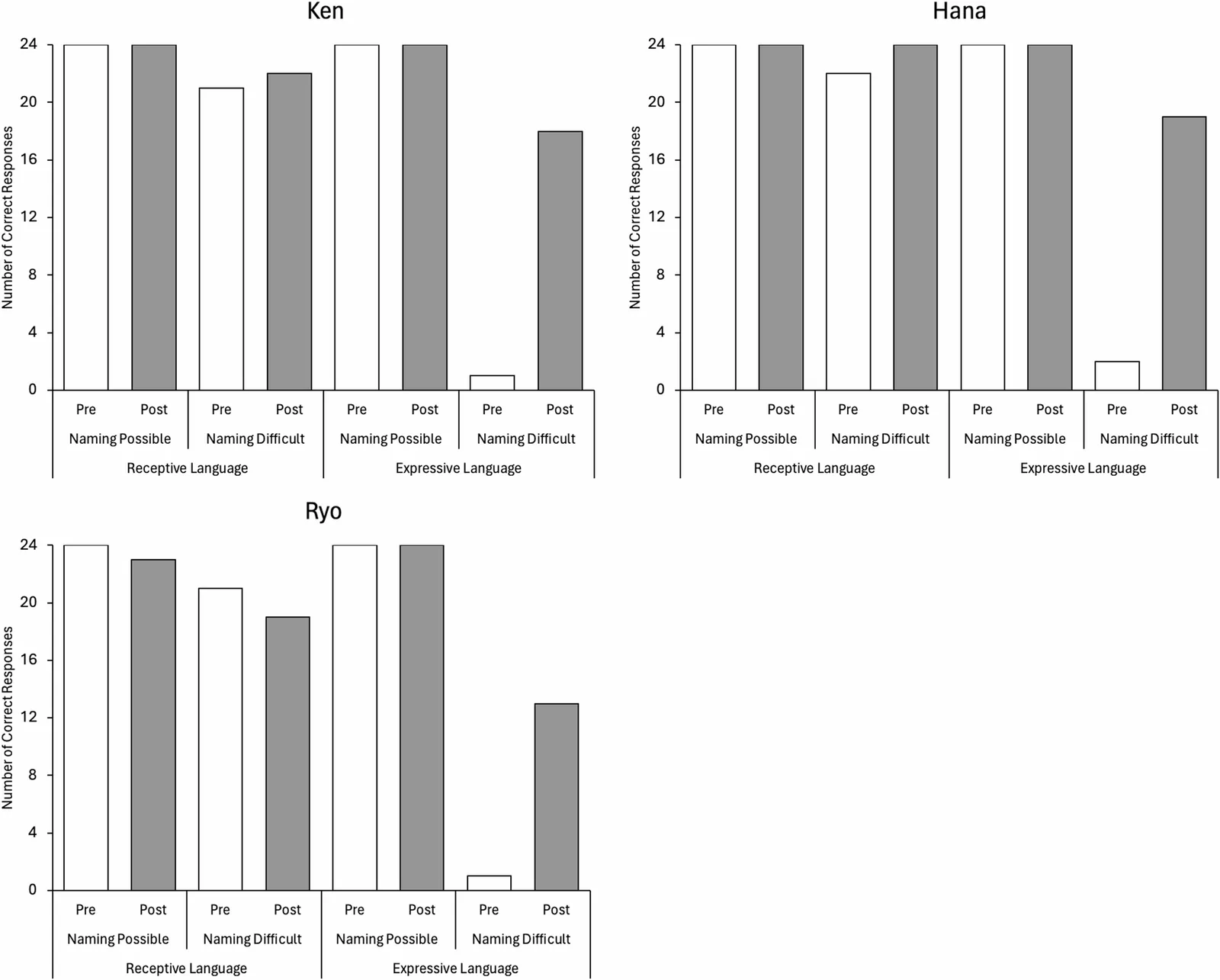
The number of correct responses in the Language Assessment in the Naming Possible Set and Naming Difficult Set

The number of correct responses for receptive and expressive language in the naming possible set and naming difficult set for each participant is shown in Fig. 3.

For Ken, in the naming possible set, the number of correct responses for receptive language was 24 in both the pre-assessment and post-assessment, as shown in Fig. 3. For expressive language, the number of correct responses was also 24 in both assessments. In the naming difficult set, the number of correct responses for receptive language was 21 in the pre-assessment and increased to 22 in the post-assessment, showing a 1-point increase. For expressive language, the number of correct responses was 1 in the pre-assessment, but it increased to 18 in the post-assessment, showing a 17-point increase.

For Hana, in the naming possible set, the number of correct responses for receptive language was 24 in both the pre-assessment and post-assessment, as shown in Fig. 3. For expressive language, the number of correct responses was also 24 in both assessments. In the naming difficult set, the number of correct responses for receptive language was 22 in the pre-assessment and increased to 24 in the post-assessment, showing a 2-point increase. For expressive language, the number of correct responses was 2 in the pre-assessment and increased to 19 in the post-assessment, showing a 17-point increase.

For Ryo, in the naming possible set, the number of correct responses for receptive language was 24 in the pre-assessment but decreased to 23 in the post-assessment, showing a 1-point decrease. The number of correct responses for expressive language remained at 24 in both assessments. In the naming difficult set, the number of correct responses for receptive language was 21 in the pre-assessment and decreased to 19 in the post-assessment, showing a 2-point decrease. For expressive language, the number of correct responses was 1 in the pre-assessment and increased to 13 in the post-assessment, showing a 12-point increase.

## Discussion

### The Impact of Removing the SGD’s Synthetic Speech Output and Delaying Reinforcer (Item) Presentation on the Frequency of Verbal and SGD Requests

In the speech output off and delayed reinforcement condition, all three participants showed an increase in the frequency of verbal requests compared to the speech output on an immediate reinforcement condition (with synthetic speech and immediate presentation).

The increase in verbal requests following the removal of synthetic speech from the SGD and the delayed presentation of reinforcers may be attributed to the delay in the reinforcer presentation, which might have caused the extinction of SGD requests. As a result, verbalization, an alternative behavior, might have emerged. In fact, in the first session of the speech output off and delayed reinforcement condition, new behaviors were observed as the SGD requests were extinguished. For example, Hana produced the utterance “OTOGA (sound)” and demonstrated arm grasping behavior after tapping the photo button on the SGD. Similarly, Ryo exhibited the behavior of pressing the side buttons on the SGD, and it was hypothesized that Ryo was manipulating the volume buttons. Moreover, all three participants exhibited repeated tapping of the photo buttons after a delay in the presentation of the reinforcer. After these new behaviors emerged, the frequency of verbal requests increased, suggesting that the extinction of SGD requests led to the emergence of verbalization as an alternative behavior.

Furthermore, for all three participants, verbal requests did not occur independently but emerged in conjunction with the use of the SGD. Specifically, verbalization was observed either after a delay following the tap of the photo button or immediately after or simultaneously with the tap. If verbalization occurred during the delay, it is likely that the absence of synthetic speech output from the SGD or the absence of item presentation served as discriminative stimuli, eliciting verbalization. Hana and Ryo also modified or added vocabulary to their verbalizations during the delay. For example, Hana tapped the picture button for the item " DOUBUTSU-NO-JIGSAW-PUZZLE (animal jigsaw puzzle)” and initially said “JIGSAW-PUZZLE,” followed by the addition of “DOUBUTSU-NO (animal)” to form “DOUBUTSU-NO-JIGSAW-PUZZLE.” Ryo, after tapping the picture button for the item “PENGIN (penguin),” initially said “HIYOKO (chick)” and then corrected the utterance to “PENGIN.” This behavior suggests that the failure of the reinforcer (the requested item) to be provided served as a discriminative stimulus, leading to a change or addition to the verbalization.

On the other hand, when verbalization occurred immediately after or simultaneously with the tap of the photo button, it is likely that the “presence of the SGD” served as a discriminative stimulus, prompting verbalization. These changes in discriminative stimuli are believed to have contributed to the reinforcement of the behavior chain, where verbalization followed the tapping of the SGD’s photo buttons in the speech output off and delayed reinforcement condition. As a result, the control of the verbal behavior shifted from being influenced by the absence of synthetic speech output or the non-provision of items to being influenced by the presence of the SGD itself. In fact, in the early sessions, all three participants exhibited verbalization more often during the delay after tapping the photo button. However, verbalization occurred more frequently over time immediately after or simultaneously with the tap. Furthermore, all three participants showed that verbalization occurred immediately after or simultaneously with the tap in the first session of the second speech output on an immediate reinforcement condition, suggesting that the “presence of the SGD” functioned as a discriminative stimulus for verbalization.

Additionally, in Ken’s case, new behaviors emerged during the speech output off and delayed reinforcement condition, including repeatedly tapping multiple photo buttons on the SGD in a clockwise motion or randomly pressing multiple buttons. It is unclear whether these behaviors were a result of the delayed presentation of the reinforcer, the absence of synthetic speech output from the SGD, or whether Ken simply enjoyed the sensory stimulation of repeatedly tapping the buttons, possibly appreciating the visual feedback (e.g., the red frame around the tapped photo button).

### Comparison of Naming Possible Set and Naming Difficult Set

#### Conditions with Synthetic Speech Output from SGD and Immediate Reinforcer (Item) Presentation (Speech Output On and Immediate Reinforcement)

In the speech output and immediate reinforcement condition, both Ken and Hana consistently made more SGD requests than verbal requests across all sessions for both the naming possible set and the naming difficult set. Conversely, Ryo made more verbal requests than SGD requests in just one session of the naming possible set, with SGD requests being more frequent in all other sessions. This suggests that, even when verbalizing the item names was an option, SGD remained the dominant communication mode during request situations.

One factor contributing to the higher number of SGD requests in the naming possible set may be the lower response effort required compared to verbal requests (Gevarter et al., 2016). Previous studies suggest that the level of response effort influences the use of AAC methods (Gevarter et al., 2016; Johnson et al., 2004), with participants potentially selecting the communication mode requiring less effort.

Another contributing factor could be the visual effects associated with the SGD, such as the red border around the photo button when tapped (van der Meer et al., 2012a; van der Meer, Sutherland, O’Reilly, Lancioni, & Sigafoos, 2012c), or the synthetic speech output produced by the SGD (Sigafoos et al., 2005; van der Meer et al., 2012a; van der Meer, Kagohara, Achmadi, O’Reilly, Lancioni, Sutherland, & Sigafoos, 2012; van der Meer et al., 2012). These inherent characteristics of the SGD may have provided higher reinforcing value. In fact, Ken exhibited behavior of randomly tapping multiple photo buttons during the second speech output and immediate reinforcement condition, suggesting he may have enjoyed the tapping action or the sensory feedback from the visual effects and synthetic speech.

Additionally, in the second speech output and immediate reinforcement condition, Ken made more verbal requests for items in the naming difficult set than in the naming possible set. This may be related to the length of the verbalization required for the requested items. The item Ken most frequently requested through verbalization in the second speech output and immediate reinforcement condition was “ROBOTTO (robot),” which had a shorter vocabulary length compared to “OUTA-EHON” (singing picture book), the most frequently requested item in the naming possible set. Therefore, the lower response effort required for the shorter utterance may have facilitated verbalization (Shillingsburg et al., 2020).

For Hana and Ryo, the frequency of verbal requests was lower in the naming difficult set compared to the naming possible set. Many items in the naming possible set required one-word utterances, while some items in the naming difficult set required two- or three-word utterances. The increased response effort associated with the longer utterances required in the naming difficult set may have reduced the frequency of verbal requests compared to the naming possible set (Shillingsburg et al., 2020). Additionally, for Ryo, some items may not have established a clear relationship between the item and the verbal stimuli, which could have contributed to the low frequency of verbal requests.

#### Conditions with Removal of Synthetic Speech from the SGD and Delayed Presentation of Reinforcer (Item) During SGD Requests (Speech Output Off and Delayed Reinforcement)

In the speech output off and delayed reinforcement condition, all three participants showed an increase in the frequency of verbal requests in the naming possible set. In the naming difficult set, although the degree of increase varied between participants and items, verbal requests also increased. This suggests that by removing the synthetic speech from the SGD and delaying the presentation of the reinforcer during SGD requests, not only were verbal responses corresponding to already learned item names promoted, but new verbal responses were also facilitated.

The increase in verbal requests for difficult-to-name items may be attributed to the influence of prior SGD training, the synthetic speech output, and the experimenter’s vocal feedback through the speech output off and immediate reinforcement condition. Previous studies have suggested that consistent SGD voice models, an increase in the number of voice models, and the simultaneous presentation of voice output with symbols on the screen may promote speech production (Kasari et al., 2014). By pairing the synthetic speech output from the SGD with the experimenter’s vocal feedback and the items, a relationship between the items and spoken language stimuli was established, facilitating an increase in the number of newly producible item names. Furthermore, in the speech output off and delayed reinforcement condition, the extinction of SGD requests may have contributed to the emergence of verbalization as an alternative behavior.

In practice, for Ken, the item “MIDORI-NO-TAIKO” (green drum) exhibited a high frequency of verbal requests in the speech output off and delayed reinforcement condition. This item had also been the most frequently requested via SGD in the first speech output on an immediate reinforcement condition. Since Ken had many opportunities to hear the voice model, it seems that verbalization corresponding to the item became feasible. On the other hand, for the item “ROBOTTO” (robot), no verbalization corresponding to the item was produced in the speech output off and delayed reinforcement condition. Instead, the utterances “KUDASAI” (please) and “KASHITE” (lend me) emerged. This could be due to the low number of requests for this item in the first speech output on an immediate reinforcement condition, which limited the opportunities to hear the voice model. As a result, the relationship between the item and the spoken language stimuli was not established, leading to the emergence of alternative behaviors such as saying “KUDASAI” and “KASHITE.”

#### Pre and Post -Language Assessment

For receptive language, in the naming possible set, Ken and Hana maintained the same number of correct responses from pre-assessment to post-assessment. In the naming difficult set, there was a slight increase in the number of correct responses. In contrast, for Ryo, the number of correct responses in the naming possible set decreased by one, and in the naming difficult set, the number decreased by two. These differences between participants suggest that the procedures in this study did not have a consistent impact on receptive language.

For expressive language, the results in the naming possible set were consistent for all three participants from pre-assessment to post-assessment. In the naming difficult set, the total number of correct responses increased significantly for all three participants in the post-assessment. The increase in the naming difficult set was likely influenced by the pairing of the SGD’s synthetic speech or the experimenter’s vocal feedback with the items, which enabled the participants to produce verbal responses corresponding to the items. Additionally, the behavior of producing item names during the speech output off and delayed reinforcement condition was reinforced. However, there were some items in the naming difficult set for which the number of correct responses did not increase significantly. Therefore, these findings suggest that the procedures in this study alone were not sufficient to enable participants to name all items.

### Clinical Significance of this Study

By removing the synthetic speech output from the SGD and combining it with delayed reinforcement for SGD requests, spoken language use was encouraged. Furthermore, even for items that were difficult to name in the pre-assessment, some cases showed facilitation of verbal requests. These results suggest that a new stimulus relationship between items and spoken language was formed through this study, expanding the repertoire of items that could be requested using speech.

The findings of this study highlight the effectiveness of a novel strategy for promoting verbal requests while utilizing SGD. Providing AAC tools such as SGDs is recommended for individuals with limited expressive language to ensure effective communication. This study demonstrated that verbal requests may be promoted while continuing to use SGD. One advantage of promoting verbal requests alongside SGD use is the potential improvement in communication efficiency. For instance, when a person desires an item within their visual field, making a spoken request may be faster and more efficient than searching for the item on the SGD screen. Additionally, since SGDs may be prone to malfunctions or battery depletion (Cagliani et al., 2019; Gevarter et al., 2016; Muharib et al., 2021), acquiring spoken communication as an alternative may help individuals adapt to various situations. Furthermore, spoken communication is the most common method (Bondy, 2012; Rush et al., 2020; Paul et al., 2013), and family members or support staff may prefer to encourage speech development.

However, in the condition where synthetic speech was output from the SGD and reinforcement was provided immediately, SGD requests were dominant, not only for items that were difficult to name but also for items that could be named. Various studies have pointed out that SGD is preferred over other AAC methods (Sigafoos et al., 2005; van der Meer et al., 2012a; Meer et al., 2012b, c). It has been suggested that preferences for such AAC tools also influence learning efficiency as a means of communication (Pak et al., 2023). Based on these studies, it can be concluded that the reason why SGD is preferred by the participants in this study is that it requires less motor effort (Gevarter et al., 2016; Sigafoos et al., 2005). Therefore, when selecting or transitioning communication modes, it is important for support staff and families to consider both the ease of use and the preferences of the individual. This approach increases the likelihood of selecting the most suitable communication mode for the participant.

Moreover, among the items deemed difficult to name in the pre-assessment, some did not show facilitation of verbal requests through the procedures of this study alone. Since the degree of facilitation of verbal requests varied among items, it is recommended that AAC tools not be prematurely discontinued, even when verbal requests are acquired for some items. Ensuring access to AAC tools, such as SGD, remains essential for maintaining communication, and utilizing multiple communication modes allows for greater adaptability across various situations and contexts.

### Limitations of this Study

The following four limitations of this study should be noted. First, due to time constraints, an ABA design was used. However, it would be better to use an ABAB design to confirm an increase in verbal requests due to speech off. Second, procedural integrity with a third party was not measured to evaluate the agreement of the procedures conducted by the experimenter. Third, there is the possibility that the reinforcing value of certain items was not sufficiently high for some participants. For example, in the case of Ryo, trials were observed where no requests were made for certain items. This suggests that the reinforcing value of the item itself may have been low, thereby suppressing the occurrence of requesting behavior. Future studies should include additional preference assessments, such as directly presenting the items to evaluate preferences and to ensure the selection of items with higher reinforcing values. Lastly, consistent results were not obtained across participants in the post-assessment of receptive language. To obtain clearer results in future research, it may be necessary to increase the sample size of participants, as well as the number of trials for receptive language assessment, to gather more data.

### Future Directions

The study was conducted in a university-based individual instruction setting, and no investigation was made into the participants’ daily life environments. While the intervention was carried out in an environment where the SGD was set up on a desk, in real-life situations, the SGD may not always be within reach or directly in front of the child when making requests. For example, if the SGD is placed farther away, the mode of communication used may change. Previous studies have reported that vocalizations were induced when the distance between the participant and the SGD was increased (Cagliani et al., 2019). Considering the potential influence of the positioning and distance of the SGD on verbal communication, it is necessary to investigate the use of SGDs in more natural life settings, such as at home or in school.

In addition, the study did not clarify the differences in the characteristics of items for which verbal requests were promoted and those for which verbal requests were not promoted. Future research should consider various variables, such as the item name’s length, the items’ reinforcing value, and the participant’s existing expressive vocabulary repertoire, and examine these differences under controlled conditions. In particular, previous studies on SGD and AAC have suggested that SGD promotes speech (e.g., Alzrayer et al., 2020; Gevarter et al., 2016), but the mechanism by which speech production is achieved remains unclear. In fact, in this study, speech output increased with the removal of synthetic speech and the delay of reinforcement presentation using SGD, but other studies (Schlosser et al., 2007; Sigafoos et al., 2003, 2011) have not observed the influence of synthetic speech. The difference between these studies is whether the reinforcement is immediate or delayed, and it is necessary to analyze the components of the intervention that can be combined with SGD.

Furthermore, Ken repeatedly tapped the SGD button during his second speech on and immediate conditions, indicating that further research is needed on this type of behavior. Andzik et al. (2022) report that stereotypic behaviors, such as repeatedly pressing the same icon, were observed in several cases of AAC electronic device use and there are limited studies on AAC-related stereotypy. Such AAC-related stereotypy may be maintained by automatic reinforcement of the outputted speech stimuli (Andzik et al., 2024) and it has been suggested that removing speech stimuli may reduce this behavior (Cook et al., 2017). Although AAC-related stereotypy was not a major research topic in this study, further research on these stereotypies is necessary for the appropriate use of SGD as a communication device.
